# A novel platelets-related gene signature for predicting prognosis, immune features and drug sensitivity in gastric cancer

**DOI:** 10.3389/fimmu.2024.1477427

**Published:** 2024-11-13

**Authors:** Qun Li, Cheng Zhang, Yulin Ren, Lei Qiao, Shuning Xu, Ke Li, Ying Liu

**Affiliations:** Department of Medical Oncology, The Affiliated Cancer Hospital of Zhengzhou University and Henan Cancer Hospital, Zhengzhou, China

**Keywords:** gastric cancer, platelets, prediction model, prognosis, gene signature, tumor microenvironment

## Abstract

**Background:**

Platelets can dynamically regulate tumor development and progression. Nevertheless, research on the predictive value and specific roles of platelets in gastric cancer (GC) is limited. This research aims to establish a predictive platelets-related gene signature in GC with prognostic and therapeutic implications.

**Methods:**

We downloaded the transcriptome data and clinical materials of GC patients (n=378) from The Cancer Genome Atlas (TCGA) database. Prognostic platelets-related genes screened by univariate Cox regression were included in Least Absolute Shrinkage and Selection Operator (LASSO) analysis to construct a risk model. Kaplan-Meier curves and receiver operating characteristic curves (ROCs) were performed in the TCGA cohort and three independent validation cohorts. A nomogram integrating the risk score and clinicopathological features was constructed. Functional enrichment and tumor microenvironment (TME) analyses were performed. Drug sensitivity prediction was conducted through The Cancer Therapeutics Response Portal (CTRP) database. Finally, the expression of ten signature genes was validated by quantitative real-time PCR (qRT-PCR).

**Results:**

A ten-gene (*SERPINE1*, *ANXA5*, *DGKQ*, *PTPN6*, *F5*, *DGKB*, *PCDH7*, *GNG11*, *APOA1*, and *TF*) predictive risk model was finally constructed. Patients were categorized as high- or low-risk using median risk score as the threshold. The area under the ROC curve (AUC) values for the 1-, 2-, and 3-year overall survival (OS) in the training cohort were 0.670, 0.695, and 0.707, respectively. Survival analysis showed a better OS in low-risk patients in the training and validation cohorts. The AUCs of the nomogram for predicting 1-, 2-, and 3-year OS were 0.708, 0.763, and 0.742, respectively. TME analyses revealed a higher M2 macrophage infiltration and an immunosuppressive TME in the high-risk group. Furthermore, High-risk patients tended to be more sensitive to thalidomide, MK-0752, and BRD-K17060750.

**Conclusion:**

The novel platelets-related genes signature we identified could be used for prognosis and treatment prediction in GC.

## 1 Introduction

Gastric cancer (GC) is one of the most prevalent cancers of the digestive tract. In 2020, there were 1.09 million new cases globally, and 0.77 million deaths due to GC, making it the 5th most common type of cancer and the 4th leading cause of cancer-related death in the world (1). GC is mostly diagnosed at advanced stages owing to its occult onset and atypical early symptoms, which is associated with a dismal overall prognosis with a 5-year survival rate of 19–31% in European and American countries and 28% in China (2). The prognosis of GC patients is not reliably predicted by conventional prognostic approaches including tumor staging systems and histopathological diagnosis, partly due to molecular heterogeneity within similar tumor stages and classifications. Alternative methods for predicting the prognosis of GC patients and directing clinical management of GC treatment are still needed to be investigated.

Beyond their well-established role in pathological thrombosis and hemostasis, platelets (PLT) are increasingly being recognized for their important roles in inflammation, tissue repair, tumor growth, and tumor metastasis (3, 4). Preclinical research has shown that PLT and tumor cells interact in both direct and indirect ways, facilitating tumor cell growth and metastasis (5, 6), immune evasion (7), and chemoresistance (8). Growing clinical data has demonstrated a strong correlation between increased PLT count and poor prognosis in cancer patients (9). Cancers with hematogenous metastases, such as breast cancer, lung cancer, hepatocellular cancer, and GC, are reported to have greater prevalence of thrombocytosis (10–13), which implies that elevated PLT count could be employed to monitor the progress of certain cancers.

Tumor-educated PLT have been shown to have a role in maintaining the primary tumor microenvironment (TME). When PLT get into contact directly with cancer cells, they could be activated and form microaggregates around tumor cells, preventing the cells from being recognized by the immune system (14). Besides, PLT may additionally produce a variety of immune-modulating molecules in a contact-independent way (15, 16), helping to maintain the microenvironments of both primary and metastatic tumors. PLT, along with other non-tumor cells and extracellular matrix, collectively contribute to the immunosuppressive TME that promotes tumor cell proliferation, aids in tumor evasion of immune surveillance, and inhibits anti-tumor immune responses (3).

With the extensive use of RNA-sequencing technology, protein profiling and functional tests, comprehensive analysis of tumor-educated PLT has progressed substantially, making PLT a potential target for cancer treatment and a promising liquid biopsy marker for treatment response monitoring and tumor progression. Nevertheless, research on the specific roles played by PLT in GC is limited. In recent years, the identification of survival-associated genes using array-based databases has been utilized for guiding individualized treatment plans for GC patients (17–19). Therefore, we collected platelet-related genes (PRGs) and developed a reliable PLT-related prognostic risk signature in GC via bioinformatics analysis. The immunological status and biological function of GC patients at high and low risk were then examined. Overall, our study indicate that the PLT-related prognostic risk signature is a reliable gene signature for the prediction of GC patients’ prognosis and may strengthen the recognition of GC pathogenesis and the exploration of novel therapeutic targets for GC patients.

## 2 Materials and methods

### 2.1 Data acquisition and processing

The transcriptome data and clinical materials of GC (n=378) were downloaded from TCGA (https://portal.gdc.cancer.gov/). The clinical features are detailed in **Supplementary Table S1**. Moreover, we selected three independent validation datasets (GSE15459, GSE62254, GSE84437) from the GEO database (http://www.ncbi.nlm.nih.gov/geo/) and obtained their normalized microarray gene expression data and clinical data. We obtained a list of 300 PRGs from previous literature (**Supplementary Data Sheet 1**) (20).

### 2.2 Identification of prognostic PRGs

After collection and preprocessing the data of GC, the Univariate Cox regression analysis was performed on the PRGs collected to identify PRGs with prognostic value (P<0.05). Genetic mutations of the prognostic PRGs were analyzed on the cBioportal online tool (https://www.cbioportal.org/) using the Stomach Adenocarcinoma (TCGA, Firehouse Legacy) dataset. Moreover, a PPI network diagram of the prognostic PRGs was constructed with the STRING database (http://string-db.org/) and graphed with the Cytoscape software (21) (version 3.7.2). Differential expression analysis for the prognostic PRGs between tumor and normal tissues was performed using the “limma” package in R (version 4.2.3) (22).

### 2.3 Construction and verification of PLT signature

The least absolute shrinkage and selection operator (LASSO) regression analysis of the candidate prognostic PRGs was performed to construct a prognostic gene signature. Then, we calculated each patient’s risk score. The calculation formula is as follows:


$$Risk\;score=\sum_{i=1}^{n}Coef\;mRNA\left(i\right)\times Expression\;mRNA\left(i\right)$$

Based on the median value of the risk score, patients in the TCGA training group were divided into high- and low-risk groups. To further verify the predictive ability of the model, three independent validation datasets (GSE15459, GSE62254, and GSE84437) were included in our study. Risk scores were calculated separately for each sample in the training cohort and the GEO validation cohorts based on the same risk formula. Based on the median risk score, we could divide the patients into two subgroups of high risk and low risk to explore the prognostic differences between the two groups. The Kaplan-Meier curves and receiver operating characteristic curves (ROCs) were constructed for the training cohort and validation cohorts.

### 2.4 Independent prognostic analysis and nomogram construction

To determine if the PLT signature may serve as a standalone predictive factor in patients with GC, we preformed multivariate Cox regression analysis. A nomogram for predicting overall survival (OS) at 1, 2, and 3 years in clinical patients was constructed using the “rms” R package based on the patient’s age, histologic grade, gender, stage and risk scores.

### 2.5 Functional enrichment analysis

We utilized the “limma” R package (22) to identify differentially expressed genes (DEGs) between the high-risk group and the low-risk group with the criteria of fold change (FC) > 2 and false discovery rate (FDR) < 0.05. To further investigate the function of the DEGs, the Gene Ontology (GO) and Kyoto Encyclopedia of Genes and Genomes (KEGG) pathways were analyzed using hypergeometric distribution testing by the “ClusterProfiler” R package (23). “circlize” R package (24) visualizes the GO and KEGG results. Finally, Gene Set Enrichment Analysis (GSEA) with the Kolmogorov–Smirnov (KS) test was performed to find enriched KEGG pathways, the ridge plot was used to present the details of GSEA via the “ggstatsplot” R package.

### 2.6 Risk model’s association with TME

The Immuno-Oncology Biological Research (IOBR) R package (25) (version 0.99.9) was used to analyze the immune features and immune cell infiltration in high- and low-risk groups. Based on the 186 TME-associated signatures in the R packet IOBR, we calculated the sample enrichment score. We assessed the expression of common immune checkpoint genes between high-risk and low-risk groups. The relationship between risk score and immune checkpoint genes was analyzed with Pearson correlation test. The CIBERSORT algorithm in the IOBR package was used for calculating the relative abundance of 22 kinds of immune cells in TCGA-GC cohort, and the ESTIMATE algorithm for calculating each sample’s matrix and immune scores.

### 2.7 Drug sensitivity analysis

The Cancer Therapeutics Response Portal (CTRP) database contains data on the sensitivity of different tumor cells to different chemotherapeutic drugs. We employed the R package “oncoPredict” (26) to calculate the sensitivity of individual GC patient to different chemotherapeutic drugs based on the gene expression data (log2(TPM + 1)). Then, the difference in the area under the dose–response curve (AUC) values between high-risk and low-risk groups was evaluated.

### 2.8 Validation of expression patterns of signature genes via the human protein atlas

The protein expression of ten signature genes in GC and normal tissues was determined using immunohistochemistry (IHC) from the Human Protein Atlas (HPA) (https://www.proteinatlas.org/), which is a valuable database providing extensive transcriptome and proteomic data for specific human tissues and cells.

### 2.9 Quantitative real-time PCR validation of signature genes

The normal human gastric epithelial cell line GES-1, and four human gastric cancer cell lines, MKN45, N87, HGC27, and KATO-3, were authenticated by STR profiling. All cell lines were cultured in RPMI-1640 containing 1% penicillin/streptomycin and 10% fetal bovine serum. Cells were grown in 5% CO2 at 37°C. Total RNA was extracted using TRIzol (TransGen Biotech, China). Complementary DNA (cDNA) was synthesized using the GoScript™ Reverse Transcription Mix and Oligo(dT) kit (Promega, United States). Real-time PCR was performed using SYBR Green PCR Master Mix (FastStart Universal SYBR Green Master, Roche). Relative gene expression levels were normalized to the levels of *GAPDH* using the ΔCt method. Each experiment was operated in technical triplicate. The amplification primer sequences of each gene are detailed in **Supplementary Data Sheet 6**.

### 2.10 Statistical analysis

R software version 4.2.3 was used to conduct the statistical analysis, and p-values and FDR q-values below 0.05 were regarded as statistically significant.

## 3 Results

### 3.1 Identification of prognosis−related PRGs in GC patients

**Figure 1:**
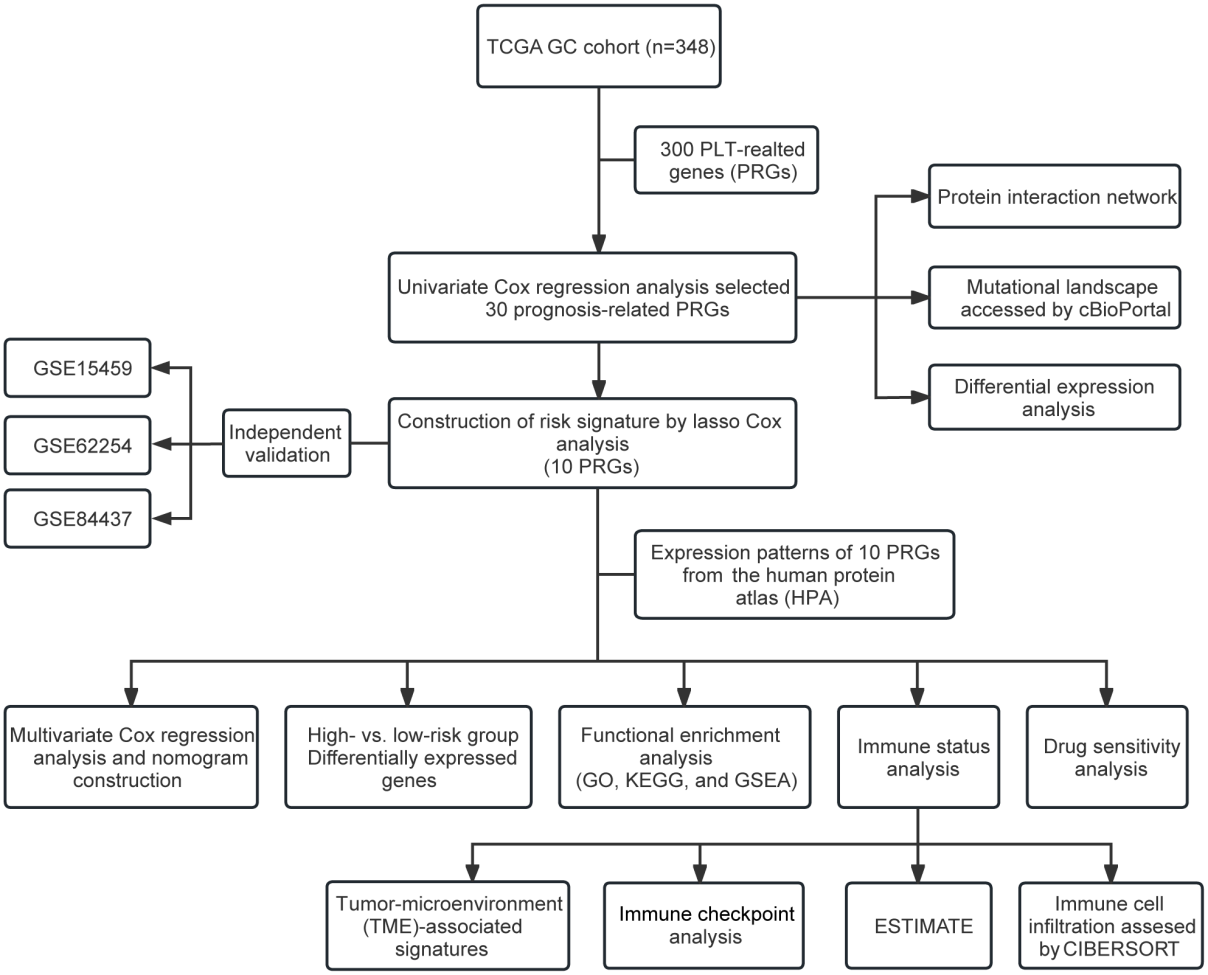
Flow chart of the study.

**Figure 2:**
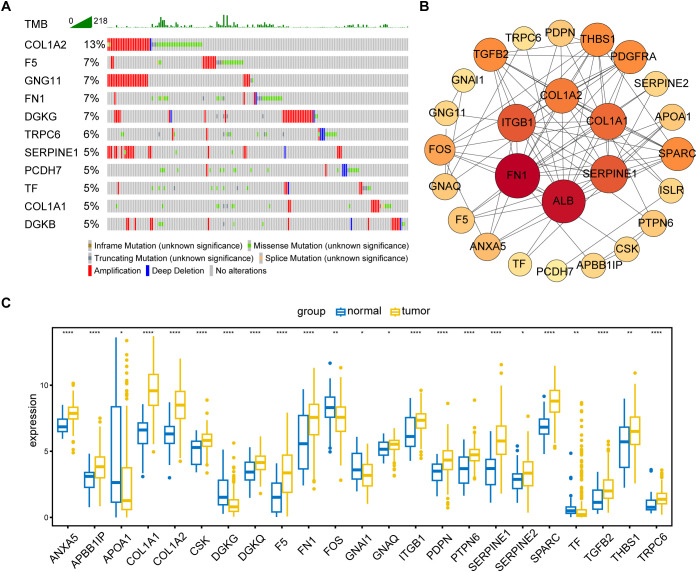
Identification and analysis of prognostic PRGs. **(A)** Gene mutation analysis of 30 prognostic PRGs in patients with GC by cBioPortal analysis (only genes altered in ≥ 5% of 478 samples are displayed). **(B)** Protein–protein interaction (PPI) network analysis of 30 prognosis-related PRGs using STRING database. **(C)** Differential expression of 30 prognostic PRGs between tumor and normal tissues (Only genes with P < 0.05 are displayed). *P < 0.05, **P < 0.01, ****P < 0.0001.

The primary design of this study was depicted in the flow chart (**Figure 1**). A total of 30 PRGs were significantly associated with prognosis of GC patients based on the Univariate Cox regression analysis (**Supplementary Data Sheet 2**). Genetic mutations of the 30 prognostic PRGs were analyzed through cBioPortal online tool for GC patients. Genes with a mutation rate no less than 5% are shown in **Figure 2A**. *COL1A2* had the highest mutation rate (13%) among 478 patients, followed by *F5* (7%), *GNG11* (7%), *FN1* (7%), and *DGKG* (7%). We constructed a protein interaction network for the 30 genes based on the STRING database. As shown in **Figure 2B**, the *FN1*, *ALB*, *SERPINE1*, *COL1A1*, *COL1A2*, and *ITGB1* genes were at the core of the protein network interaction. The differential expression analysis of the candidate prognostic PRGs revealed 23 differentially expressed genes, including 18 upregulated and 5 down-regulated genes (**Figure 2C**).

### 3.2 PLT signature establishment

**Figure 3:**
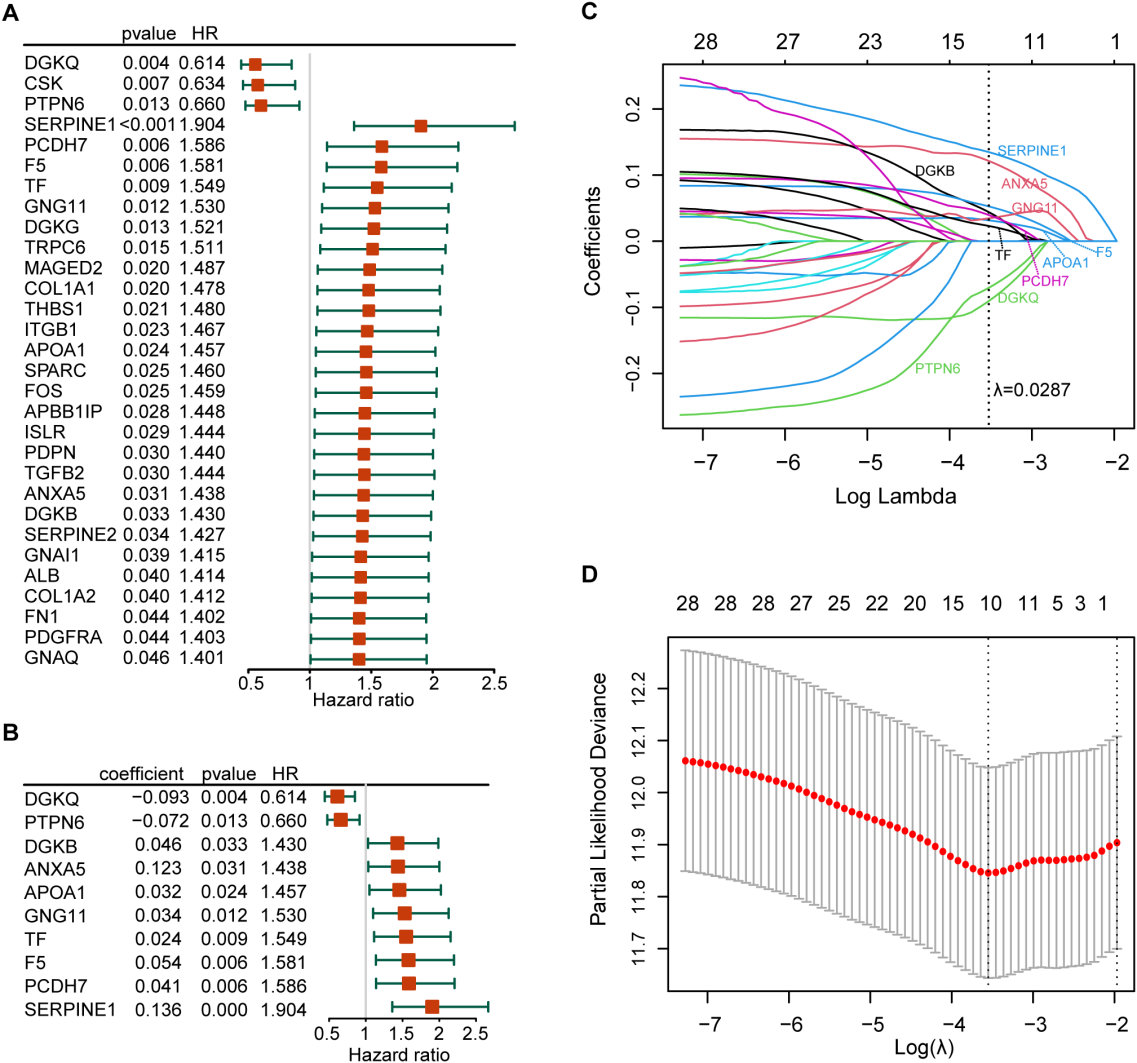
Establishment of the risk model. **(A)** The forest plot of the 30 prognosis-related PRGs obtained by univariate Cox regression analysis; **(B)** Construction of prognostic signatures based on lasso Cox analysis; **(C)** LASSO coefficient profiles of 30 prognosis-related PRGs, genes are represented by different colors; **(D)** LASSO regression with tenfold cross-validation, and selection of the optimal parameter (lambda) in the LASSO model.

The forest plot of the 30 prognostic PRGs obtained by univariate Cox regression analysis was shown in **Figure 3A**. Then we constructed a predictive prognostic model consisting of 10 PRGs by LASSO regression analysis (**Figures 3B–D**). They were *SERPINE1*, *ANXA5*, *DGKQ*, *PTPN6*, *F5*, *DGKB*, *PCDH7*, *GNG11*, *APOA1*, and *TF*. The coefficient and HR value of multivariate Cox regression analysis is shown in the form of a forest map (**Figure 3B**). A linear prediction model was developed based on the weighted regression coefficients of 10 prognostic PRGs, calculated as $\begin{array}{l} risk\;score=(-01357\times SERPINE1\;\exp)+(0.12273\times ANXA5\;\exp)+(-0.0927)\times \\ DGKQ\;\exp+(-0.0722\times PTPN6\;\exp)+(0.05354\times F5\;\exp)+(0.04579\times DGKB\;\exp)+\\ (0.04079\times PCDH7\;\exp)+(0.03418\times GNG11\;\exp)+(0.03202\times APOA1\;\exp)+\\ \left(0.02374\times TF\;\exp\right) \end{array}$ . Of these, *SERPINE1*, *ANXA5*, *F5*, *DGKB*, *PCDH7*, *GNG11*, *APOA1*, and *TF* showed significant positive correlations with risk scores, while *DGKQ* and *PTPN6* showed significant negative correlation with risk scores.

### 3.3 Validation of the PLT signature

**Figure 4:**
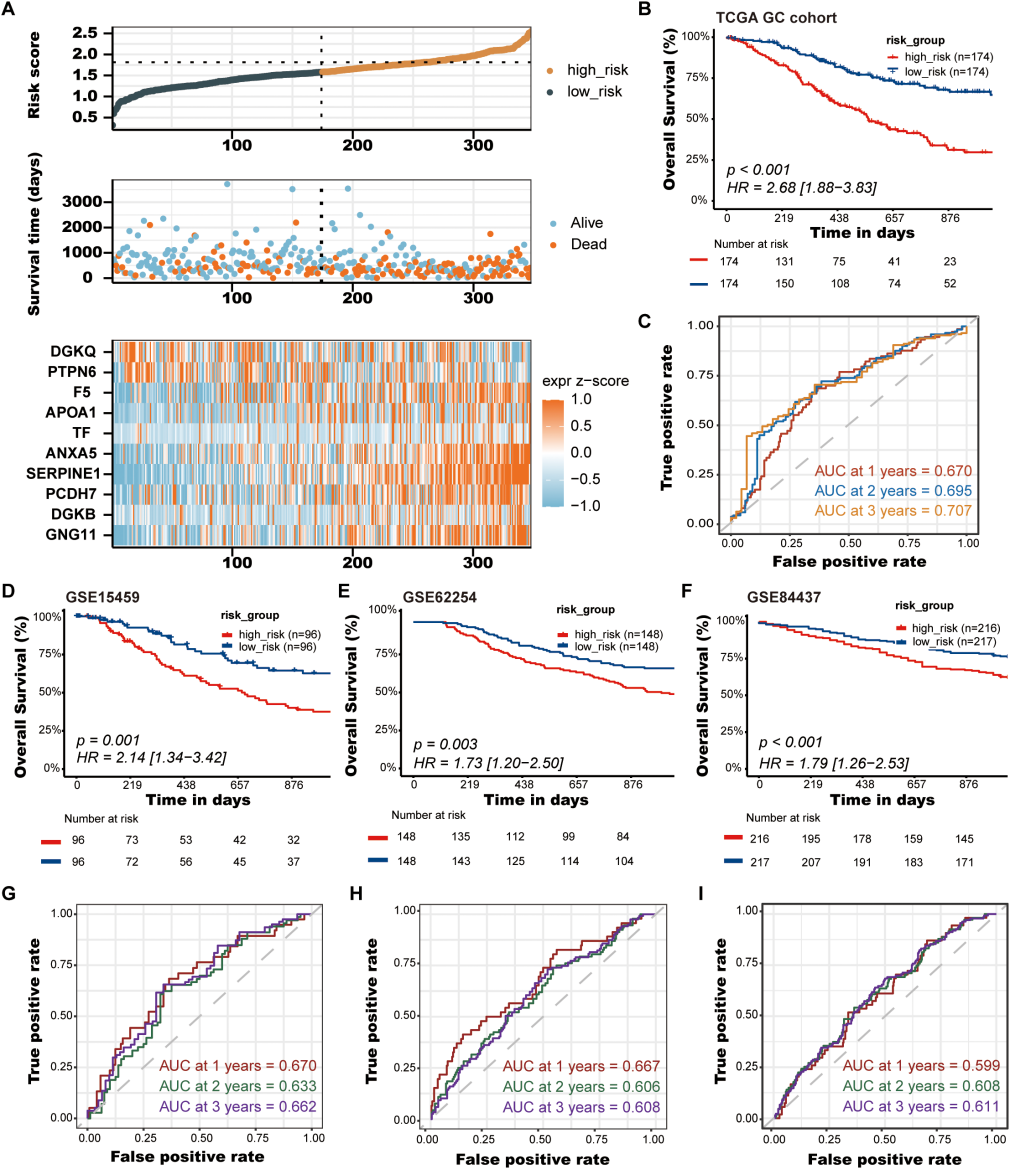
Validation of the PLT signature. **(A)** The distribution of risk score, the survival status and the heatmap of GC patients in the TCGA cohort; **(B)** Kaplan-Meier survival curves of OS between low-risk and high-risk groups in the TCGA cohort; **(C)** Time-dependent ROC curves of 1-, 2-, and 3-years of GC patients in TCGA cohort; **(D-F)** Kaplan-Meier survival curves of OS between low-risk and high-risk groups in the GSE15459, GSE62254, and GSE84437 cohorts, respectively; **(G-I)** Time-dependent ROC curves of 1-, 2-, and 3-years of GC patients in the GSE15459, GSE62254, and GSE84437 cohorts, respectively.

After establishing the predictive prognostic model based on 10 prognostic PRGs for GC, we computed the risk score for each GC patient based on the LASSO coefficients and expression value for each PRG (**Supplementary Data Sheet 3**). We contrasted the distribution of risk score, the survival status and the heatmap of GC patients in the TCGA cohort (**Figure 4A**). The risk curves and scatter plots implied that mortality was positively related to the risk score in the TCGA cohort. The heatmap unveiled that higher *DGKQ* and *PTPN6* expression were detected in the low-risk group, while the other eight genes (*F5*, *APOA1*, *TF*, *ANXA5*, *SERPINE1*, *PCDH7*, *DGKB*, and *GNG11*) were highly expressed in the high-risk group. Kaplan-Meier analysis was used to analyze the survival and prognosis of GC patients in TCGA. As shown in the **Figure 4B**, patients in the low-risk group had a better prognosis, while patients in the high-risk group had a worse prognosis (P<0.001). The AUCs of 1-year, 2-year, and 3-year survival ROC curves predicted by the PLT signature were 0.670, 0.695, and 0.707, respectively, suggesting the efficiency of PLT signature in predicting prognosis for GC to a certain extent (**Figure 4C**).

To further demonstrate the stability and reliable generalization of our model, the GSE15459, GSE62254, and GSE84437 cohorts were used as the external validation cohorts. The Kaplan-Meier curves showed a significant difference in prognosis between the high-risk and low-risk patients in these three cohorts, respectively, with a more significant survival advantage for patients in the low-risk group (P = 0.001, P = 0.003, P < 0.001, respectively) (**Figures 4D–F**). The ROC curve was used as a tool to predict the survival time of patients at 1-, 2-, and 3- years. The AUCs at 1-, 2-, and 3- years for the GSE15459 cohort were 0.670, 0.633, and 0.662, respectively (**Figure 4G**). The AUCs for the GSE62254 cohort were 0.667, 0.606, and 0.608, respectively (**Figure 4H**). The AUCs for the GSE84437 cohort were 0.599, 0.608, and 0.611, respectively (**Figure 4I**). This indicates that the model has an excellent predictive effect.

### 3.4 Creation of nomograms based on PLT signatures combined with clinical characteristics

**Figure 5:**
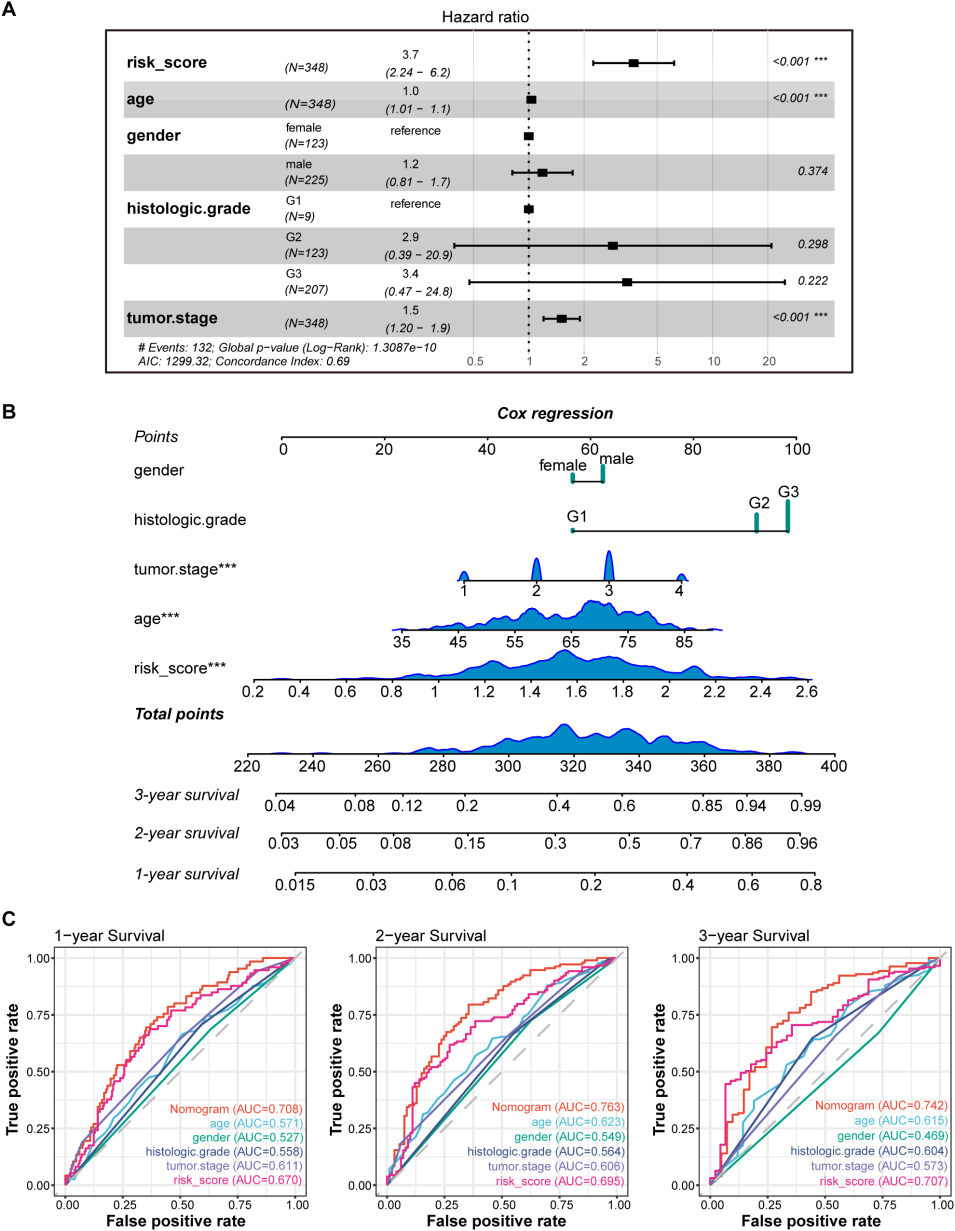
Construction and validation of the nomogram model. **(A)** Multivariate Cox analyses indicated that risk score was an independent prognostic factor significantly associated with OS in TCGA cohort; **(B)** Nomogram for predicting 1-, 2-, and 3-year OS; **(C)** Time-dependent ROC curve analyses of the nomogram, risk score, age, gender, histologic grade and tumor stage in TCGA cohort. *** P < 0.001.

To validate the reliability and clinical value of the biological signature constructed based on PRGs as a predictor of prognosis, we conducted multivariate Cox regression analysis including common clinical characteristics (**Supplementary Data Sheet 4**). It is shown that in the multifactorial cox analysis, tumor stage (P<0.001) and risk score (P<0.001) were all independent prognostic factors significantly associated with patient prognosis (**Figure 5A**). Based on the above analysis, in order to be able to predict patients’ prognosis quantitatively and to inform clinical decision-making, we integrated the risk score and clinical indicators to construct Nomogram plots as a means of predicting the probability of prognostic survival at 1, 2, and 3 years (**Figure 5B**). We then used time-dependent ROC curve analysis to compare the predictive accuracy between the nomogram, risk score, and common clinicopathological features (**Figure 5C**). The results showed that risk score had a much greater AUC value than the rest of the individual clinicopathological features, and the nomogram model suggested higher prognostic accuracy at 1-, 2-, and 3-year OS with a larger AUC than risk score. The time-dependent AUCs of the nomogram for predicting 1-, 2-, and 3-year OS were 0.708, 0.763, and 0.742, respectively. Combined with these results, this suggests that our PLT signature is more practical and influential for clinical decision making and is more suitable as a clinical decision tool for predicting the prognosis of patients with GC in the clinical setting.

### 3.5 Identification of DEGs between high-risk and low-risk groups and function enrichment analysis

**Figure 6:**
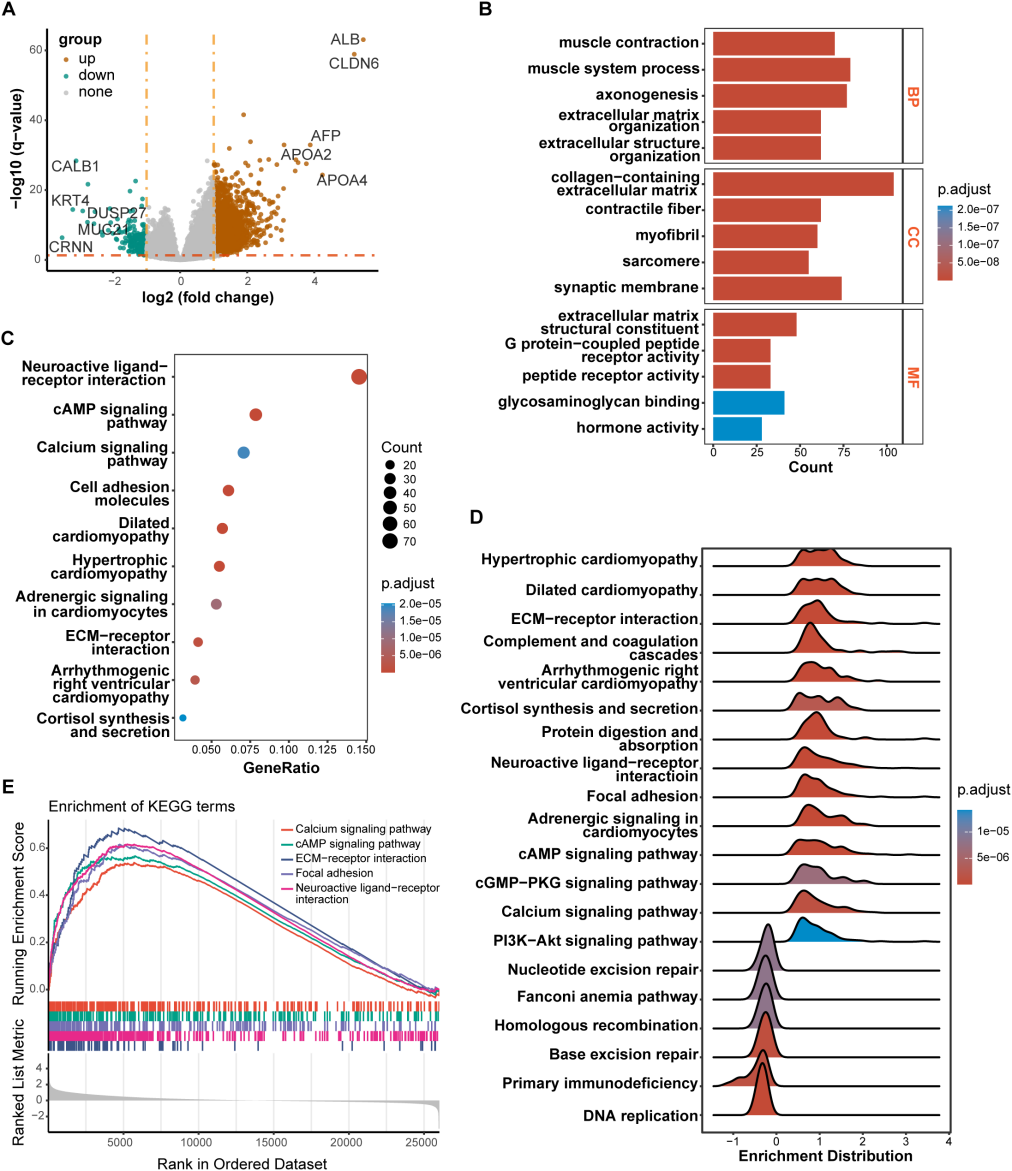
Results of differentially expressed genes (DEGs) and function enrichment. **(A)** Volcano plot of DEGs between high-risk and low-risk groups; **(B)** GO enrichment analysis of DEGs between two groups; **(C)** KEGG enrichment analysis of DEGs between two groups; **(D)** Ridgeplot of KEGG by GSEA. **(E)** GSEA plot of important pathways in comparison between two groups.

We performed DEGs analysis between high-risk and low-risk groups on the TCGA cohort, and the results showed that 2,442 DEGs were differentially expressed between the high-risk group and the low-risk group based on the criteria of P < 0.05. Among that, 2,249 genes were up-regulated, and 193 genes were down-regulated. The volcano plot of DEGs were displayed in **Figure 6A**. All of the upregulated and downregulated genes were demonstrated in **Supplementary Data Sheet 5**. The results of GO analysis can be divided into three categories: biological process, cellular component, and molecular function. Where in biological processes, such as axonogenesis, extracellular matrix organization, extracellular structure organization; Cellular components, such as collagen−containing extracellular matrix and synaptic membrane; And molecular functions, such as extracellular matrix structural constituent, G protein−coupled peptide receptor activity, peptide receptor activity, and glycosaminoglycan binding were significantly enriched (**Figure 6B**). KEGG pathways were enriched in Neuroactive ligand−receptor interaction, cyclic adenosine monophosphate (cAMP) signaling pathway, Calcium signaling pathway, Cell adhesion molecules, and extracellular matrix (ECM)−receptor interaction (**Figure 6C**). Then, the GSEA method was applied to identify the significantly enriched KEGG pathways in the high-risk samples. The ridgeplot showed that several pathways, such as calcium signaling pathway, cAMP signaling pathway, ECM-receptor interaction, focal adhesion, and neuroactive ligand-receptor interaction, were significantly enriched in the high-risk group (**Figure 6D**). DNA replication, base excision repair, homologous recombination, nucleotide excision repair were the pathways that were substantially enriched in the low-risk group. GSEA plot of important pathways enriched in the high-risk group was shown in **Figure 6E**.

### 3.6 Immune signatures between high-risk and low-risk groups

**Figure 7:**
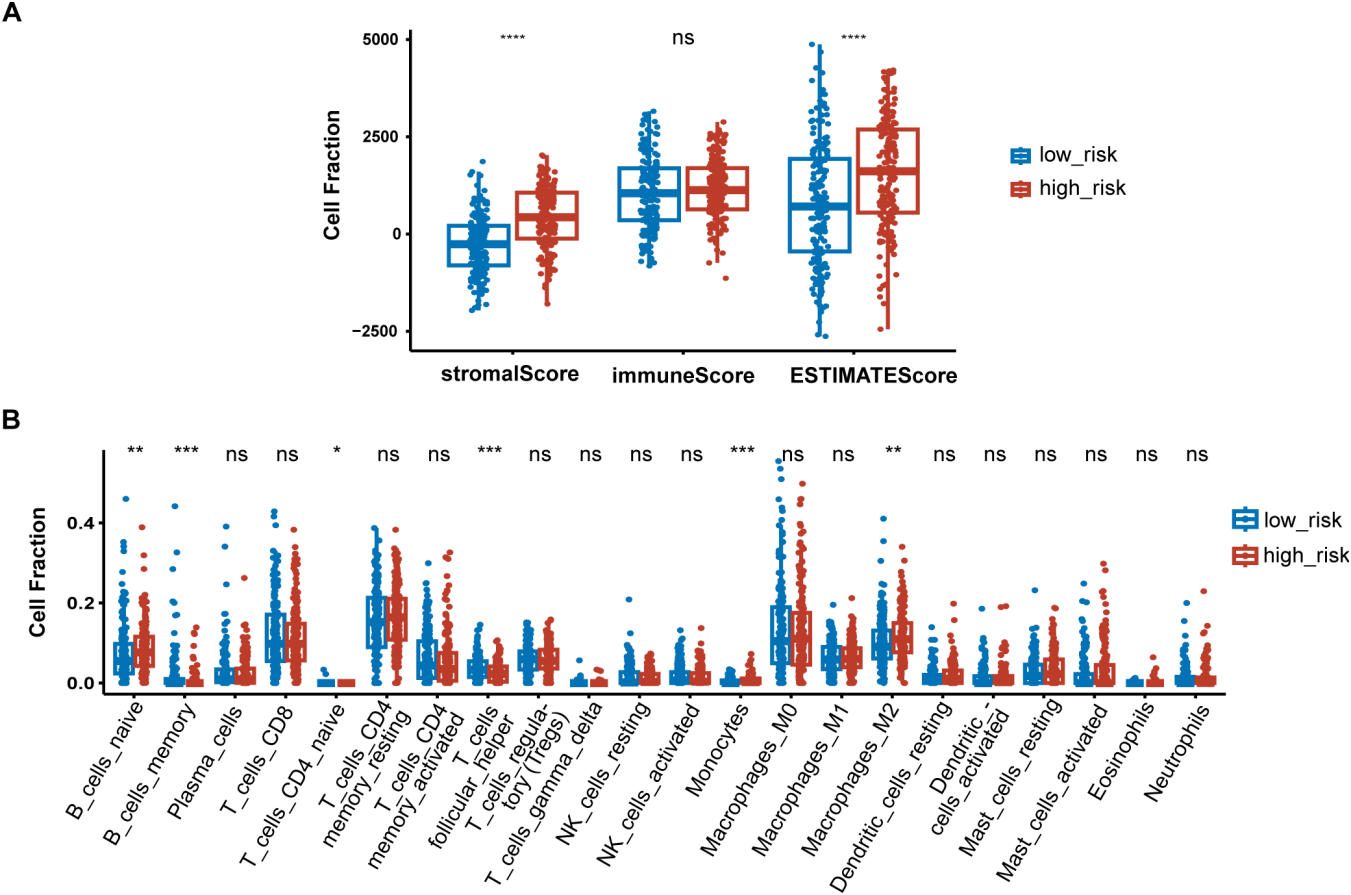
Immune signatures between high-risk and low-risk groups. **(A)** Difference of ESTIMATE immune infiltration between different risk groups in the TCGA cohort; **(B)** The proportion of immune cell components assessed by CIBERSORT in the TCGA cohort. *P < 0.05, **P < 0.01, ***P < 0.001, ****P < 0.0001, ns, not significant.

To further elucidate differences in the immune microenvironment of patients between high-risk and low-risk groups, we compared the enrichment scores of TME cells-related signatures between two groups. The results showed that T cell-related signatures [T cell accumulation, T cell exhaustion, T cell regulatory (27)] and tumor-associated macrophage-related signatures [Macrophages Bindea et al. (28), TAM_Peng_et_al (27)] had significantly higher enrichment scores in the high-risk group compared to the low-risk group (**Supplementary Figure S1**). Additional examination of TME signatures employing the IOBR package unveiled an immunosuppressive, exclusive, and exhausted TME in the high-risk group (**Supplementary Figures S2A–C**). Furthermore, patients in the low-risk group exhibited higher scores in DNA damage response (DDR), mismatch repair, and homologous recombination (**Supplementary Figure S2D**), suggesting that they may be more sensitive to immunotherapy. High-risk patients demonstrated more pronounced epithelial-mesenchymal transition (EMT) signatures (**Supplementary Figure S2E**). Taken together, these findings suggest an immunosuppressive TME in the high-risk group. The extent of immune cell infiltration in patients in the TCGA cohort was then assessed. The results of ESTIMATE suggested that stromal score, and ESTIMATE score were higher in the high-risk group (**Figure 7A**). We then estimated the proportion of 22 types of immune cells in each sample by CIBERSORT algorithm. The difference in the proportion of each type of immune cell between two risk groups was shown **Figure 7B**. The results revealed that compared with the low-risk group, memory B cells (P < 0.001), follicular helper T cells (P < 0.001) exhibited lower infiltrating levels in the high-risk group. However, samples in the high-risk group had a significant increase in the fraction of naïve B cells (P < 0.01), monocytes (P < 0.001) and macrophages M2 (P < 0.01). We also explored the relationship between risk score and common immune checkpoint genes, including programmed cell death 1 (PDCD1), PDCD1 ligand 1 (PDCD1L1/CD274), cytotoxic T-lymphocyte-associated antigen 4 (CTLA4), PDCD1 ligand 2 (PDCD1LG2), hepatitis A virus cellular receptor 2/T-cell immunoglobulin mucin receptor 3 (HAVCR2/TIM3), lymphocyte activating 3 (LAG3), and T cell immunoreceptor with immunoglobulin and ITIM domain (TIGIT). As displayed in **Supplementary Figure S3A**, the levels of HAVCR2 and PDCD1LG2 were increased in the high-risk group in GC. However, no significant correlation was observed between the immune checkpoint molecules and the risk score (**Supplementary Figure S3B**).

### 3.7 Relationship between risk scores and response to chemotherapy

**Figure 8:**
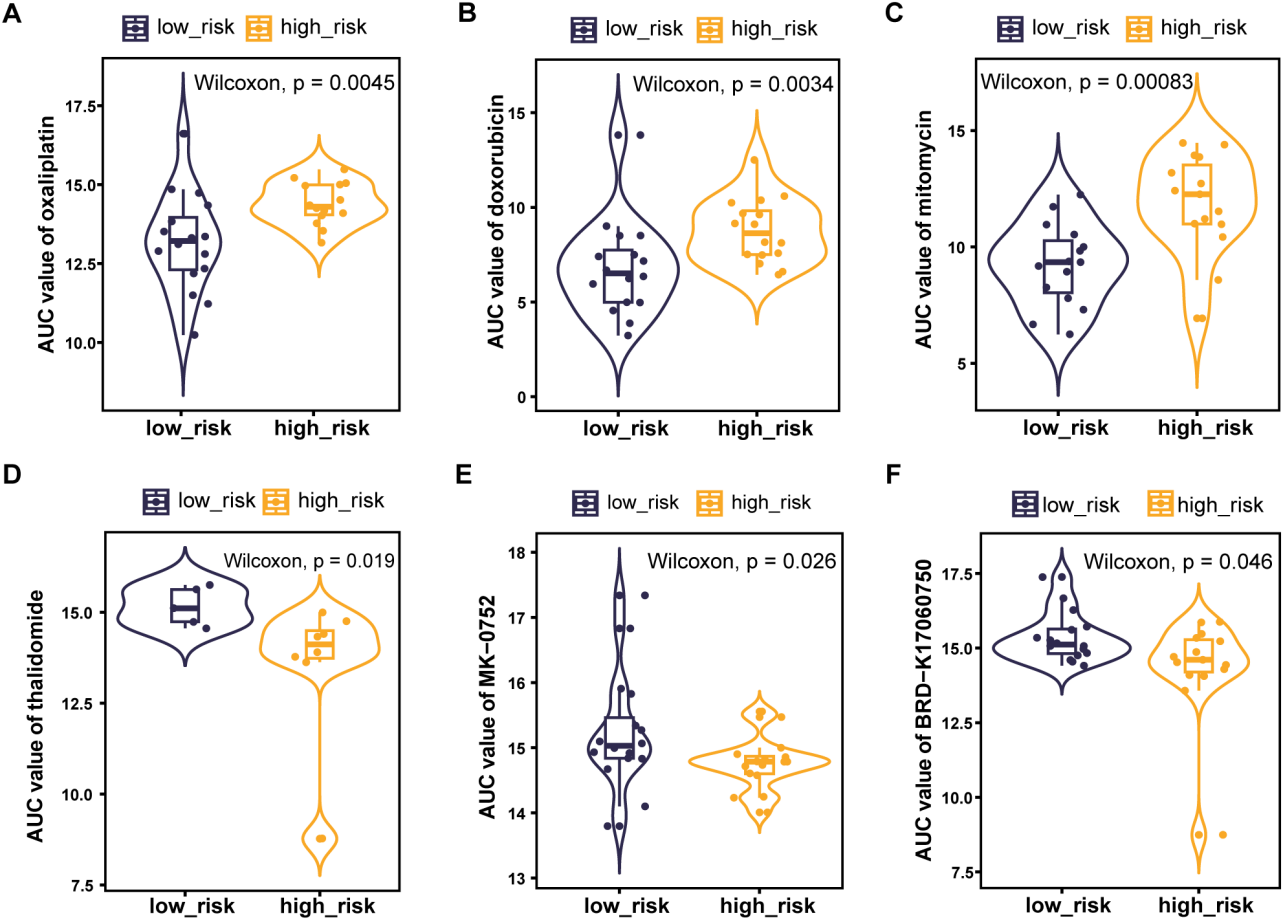
PLT signature predicts the sensitivity of chemotherapy. **(A)** Oxaliplatin; **(B)** Doxorubicin; **(C)** Mitomycin; **(D)** Thalidomide; **(E)** MK-0752; **(F)** BRD-K17060750.

In order to find more effective drugs for patients in the high-risk group, we further studied the sensitivity of tumor cells to chemotherapeutic drugs between different risk groups based on CTRP database. The AUC value represents the degree of drug sensitivity. An increasing AUC value represents a lower drug sensitivity. We found that patients in the high-risk group tended to be less sensitive to oxaliplatin, doxorubicin, and mitomycin, but more sensitive to thalidomide, MK-0752, and BRD-K17060750 (**Figure 8**).

### 3.8 Validation of the expression levels of signature genes in clinical samples

**Figure 9:**
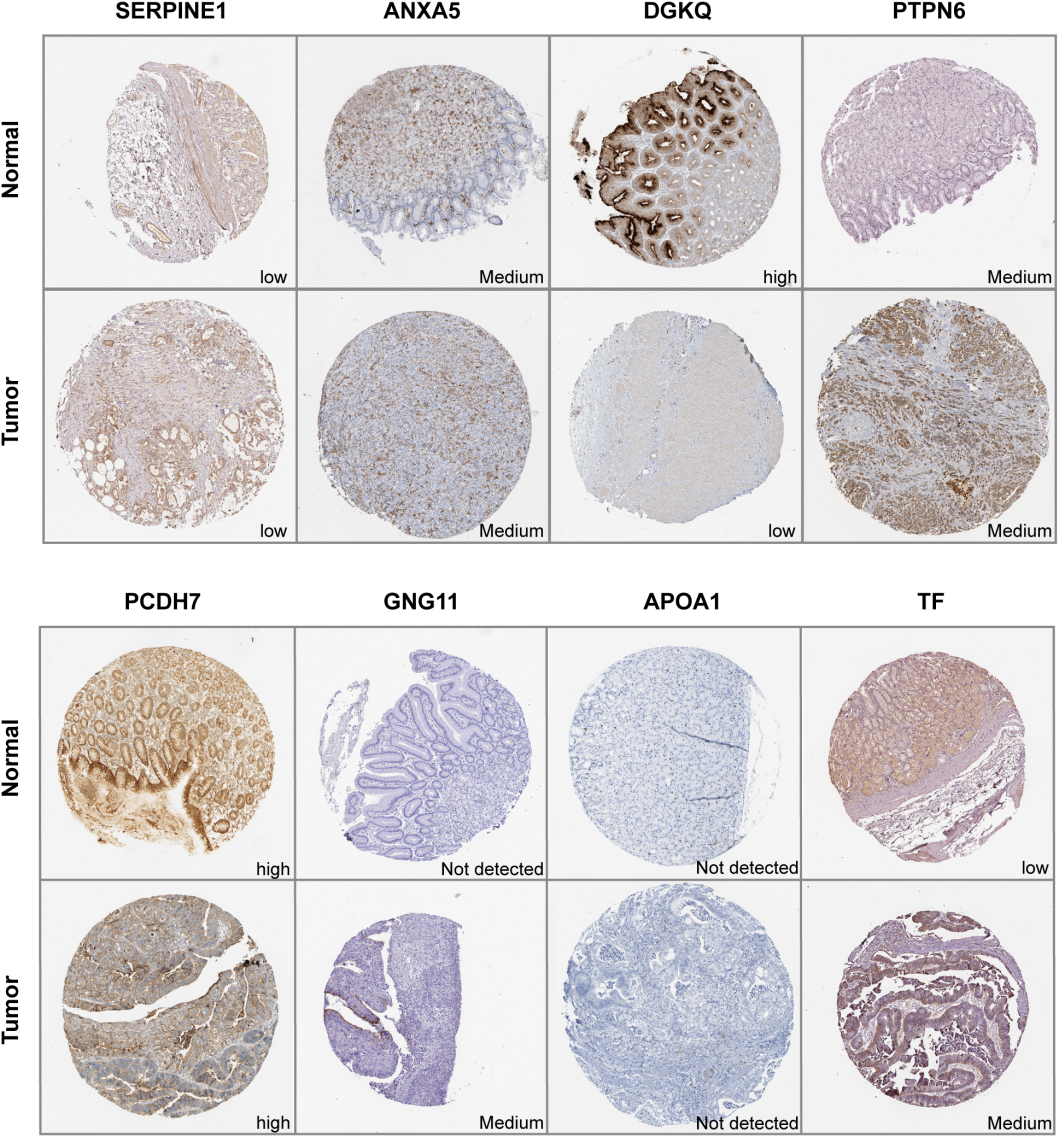
Immunohistochemistry of signature genes in GC and normal samples from the HPA database.

IHC results of the protein expression of the signature genes from HPA database were displayed in **Figure 9**. SERPINE1 was expressed at a low level in stomach normal tissues and was not detected or expressed at a low level in tumor tissues. ANXA5 was expressed at a low to medium level in both tumor and stomach normal tissues. DGKQ was not detected or expressed at a low level in GC tumor tissues but had medium to high expression levels in stomach normal tissues. PCDH7 was expressed at a high level in stomach normal tissues and had diverse expression levels in GC tumor tissues, ranging from low expression and medium to high expression. GNG11 was not detected in stomach normal tissues but had various expression levels in GC tumor tissues, which was from not detected and low to medium expression. APOA1 was not detected in both tumor and stomach normal tissues. TF was not detected or expressed at a low level in normal tissues and had diverse expression levels in GC tumor tissues, which was from not detected and low expression to medium expression. Data for F5 and DGKB were lacking and therefore not presented.

### 3.9 Validation of ten signature PRGs by qRT-PCR

**Figure 10:**
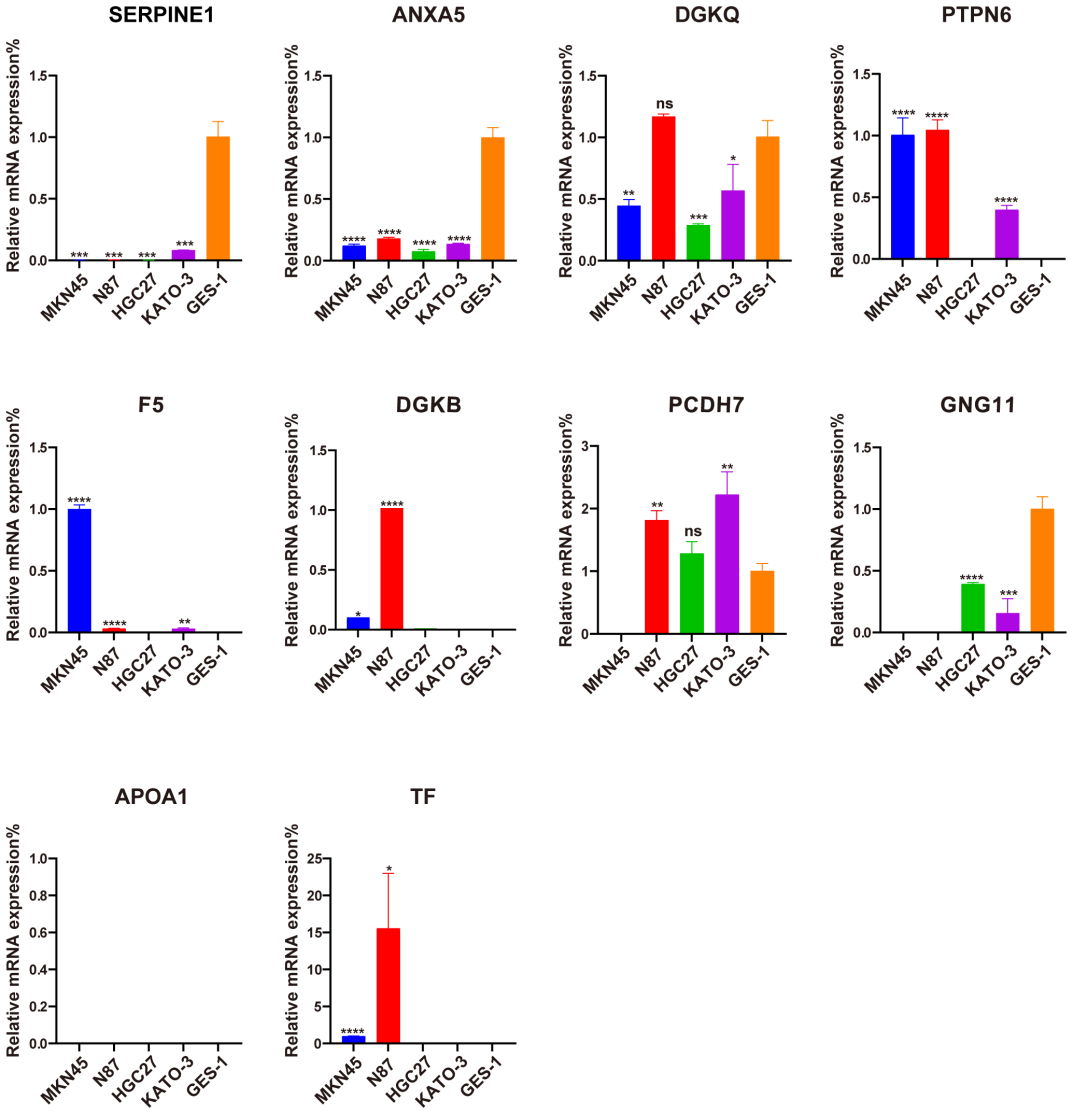
Evaluation of the expression of ten PLT-related signature genes in normal and GC cells. *P < 0.05, **P < 0.01, ***P < 0.001, ****P < 0.0001, ns, not significant.

The qRT-PCR analysis was performed to further verify ten signature genes in normal and tumor cells (**Figure 10**). The results showed that the expression of PTPN6, F5, DGKB, PCDH7, and TF was elevated in GC cell lines, whereas the expression of SERPINE, ANXA5, DGKQ, and GNG11 was decreased. APOA1 expression was not detected in the GES-1 and four human gastric cancer cell lines.

## 4 Discussion

GC is one of the most common primary malignant tumors in the digestive tract with high rates of incidence and mortality. Despite recent advances in immunotherapy and molecular targeted therapies, the prognosis of advanced GC patients is still miserably poor. The TNM staging system used by the American Joint Committee on Cancer (AJCC) is a major factor influencing prognosis and treatment decisions of GC (29). The Asian Cancer Research Group (ACRG) newly proposed a molecular classification system where GC is divided into four subtypes: microsatellite stable (MSS)/TP53 activation, MSS/TP53 loss, microsatellite instability (MSI), and MSS/EMT. The result of survival analysis demonstrated that the MSS/EMT group had the worst prognosis due to its easy metastasis and the MSI group had a better prognosis (30). Nonetheless, the existing prognostic stratification systems are not sufficient to accurately predict the prognosis in GC patients. Hence, it is still urgently necessary to explore novel and effectual molecular prognostic biomarkers for GC.

In recent years, PLT in cancers have gotten wide attention due to its roles in regulating tumor proliferation, metastasis and TME through several mechanisms (5, 7). PLT can secrete growth factors like epidermal growth factor (EGF) and vascular endothelial growth factor (VEGF), promoting tumor cell proliferation and angiogenesis (4). PLT also release transforming growth factor-β (TGFβ) and serotonin, creating an immunosuppressive microenvironment by suppressing T cell activity and promoting the transition of M1 macrophages to the M2 phenotype (31). Additionally, PLT facilitate EMT, increasing the invasiveness of tumor cells (5). Moreover, PLT can form microaggregates around circulating tumor cells, protecting them from immune detection and enhancing their ability to metastasize (32). It has been reported that PLT reduction was associated with improved OS and progression-free survival (PFS) rates in patients with stage IV GC (33). However, the effect of PLT-related mRNAs in GC and the mechanism of how PLT alterations affect the tumor biological processes of GC remains unclear to date.

In this study, we integrated PLT-related gene expression profiles from the TCGA-GC dataset and screened 10 genes to construct a new prognostic model for GC patients using LASSO regression analysis. The PLT signature we constructed was shown to be an independent prognostic factor for GC, and a substantial prognostic difference was discovered between the high and low risk groups. Furthermore, the HR of the risk score in multivariate Cox analyses was 3.7 (2.24-6.2), while the HR of tumor stage was only 1.5 (1.20–1.9). Risk score seems more pronounced than tumor stage in prognosis prediction of GC. A nomogram integrated with age, gender, histologic grade, tumor grade and risk score also showed a good prediction of GC patients’ survival in 1-, 2-, and 3- years. It helps improve clinicians’ decision-making and optimize the personalized treatment plans of GC patients. ROC curves demonstrated the PLT signature’s superiority to the other traditional clinical indicators such as age, gender, histologic grade, and tumor grade.

After a comprehensive review of the literature, we reviewed the roles of the signature genes in platelet function and their relationship with cancer, highlighting that most genes included in the PLT risk model are closely associated with cancer to varying extents. *Serine protease inhibitor clade E member 1* (*SERPINE1*) plays key roles in regulating the fibrinolytic system (34). It has been detected in various cancers and implicated in tumor progression and angiogenesis in multiple cancer types (35–37). It was reported that *SERPINE1* contributes to tumor proliferation, invasion and migration by regulating EMT in GC (38). *Annexin A5* (*ANXA5*) was identified as an anticoagulant protein and soon reported as a potential apoptosis biomarker due to its binding to phosphatidylserine (39). *ANXA5* contributes to an immunostimulatory profile in the TME and serves as a link between the innate and adaptive immune systems (39). *ANXA5* may potentially affect the prognosis of GC patients as well as the immune therapy response due to its influence on the angiogenesis phenotype (40). *Diacylglycerol Kinase Beta* (*DGKB*) and *Diacylglycerol Kinase Theta* (*DGKQ*) encode different isotypes of Diacylglycerol kinases, which regulates the intracellular concentration of the second messenger diacylglycerol. Inhibition of diacylglycerol kinase augmented platelet secretion and aggregation (41). Diacylglycerol pathways influence the tumor ecosystem by mediating the intricate and dynamic interactions between cancer cells and the tumor immune environment (42). The protein encoded by *Protein Tyrosine Phosphatase Non-Receptor Type 6* (*PTPN6*) is a member of the protein tyrosine phosphatase family (43). *PTPN6* has been shown to inhibit platelet apoptosis and necroptosis during sepsis (44), and its elevated expression is linked to poor prognosis and increased immune infiltration in cancer (45). *Coagulation factor V* (*F5*) plays an essential role in coagulation as both a procoagulant cofactor and an anticoagulant cofactor (46). High *F5* expression was associated with aggressive tumors, but also with improved survival in breast cancer (47). *Protocadherin 7* (*PCDH7*) belongs to the cadherin superfamily and plays a role in the pathways of platelet activation, signaling, and aggregation (48). Zhou et al. found that *PCDH7* could suppress cell migration and invasion through E-cadherin inhibition in GC cell lines (49). *G Protein Subunit Gamma 11* (*GNG11*) is a member of guanine nucleotide-binding protein gamma family, which regulated G-protein coupled receptors-dependent platelet function (50). Jiang et al. discovered that high expression of *GNG11* was associated with poor prognosis of ovarian cancer patients (51). *Apolipoprotein A1* (*APOA1*) encodes apolipoprotein A-I, which was shown to inhibit platelet activation and reduce both clot strength and stability *in vivo* (52). A preclinical study showed that reduced plasma APOA1 level is associated with gastric tumor growth in mouse cancer xenograft model (53). *Transferrin* (*TF*) encodes iron binding transport proteins (54). Although iron metabolism has been reported closely related to cancer progression (55), the role of *TF* in cancer has yet to be investigated.

We found that patients in the high-risk group had significantly higher activities of calcium signaling pathway, cAMP signaling pathway and ECM-receptor interaction. Ca2+ signaling is closely implicated in platelet function. Release of Ca2+ from the dense tubular system into the cytosol initiated by activated PLCγ2 can amplify platelet activation (56). It is becoming evident that dysregulated Ca2+ homeostasis may serve an important role in carcinogenesis or tumorigenesis (57). cAMP is recognized for its significant role in regulating platelets, and platelet activators are known to disrupt the cAMP signaling pathway at various levels (58). Cancer cells, including glioblastoma, ovarian cancer, colorectal cancer and breast cancer, utilize the cAMP/PKA signaling pathway to facilitate invasion, migration, adhesion, clonal development, and other malignant traits (59). Deregulation of ECM remodeling, characterized by excess matrix deposition and increased stiffness, is associated with bone marrow pathologies that can lead to defects in platelet production and function (60). ECM-receptor interaction pathway plays an essential role in tumor shedding, adhesion, and mobility (61). It has been demonstrated that in GC, ECM -receptor interaction pathway takes involvement in the process of tumor invasion and metastasis (62). Taken together, the enrichment of these pathways demonstrates to some extent the mechanism of poorer prognosis in patients with higher risk scores.

As tumor-educated PLT play significant roles in modulating the immune environment, we further explored the immune features of high-risk patients. Immune-related gene signature suggest an immunosuppressive TME in the high-risk group. Given that immune checkpoint inhibitors are less effective in an immunosuppressive microenvironment (63), we speculated that patients in the high-risk group would benefit less from immunotherapy. We further investigated the relationship between the risk scores and immune checkpoint molecules, which have been considered potential biomarkers of response to ICIs. Although the expression of HAVCR2 and PDCD1LG2 was elevated in the high-risk group, the correlations between the immune genes and the risk score were not significant. Additional biomarkers, such as tumor mutation burden (TMB) and human leukocyte antigen (HLA), merit additional investigation to gain a more comprehensive understanding of the potential relationship between the risk score and efficacy of immunotherapy.

The investigation of immune cell infiltration in different risk groups of GC patients can help clinicians to gain a better knowledge of the overall immune landscape of patients. Our findings demonstrated that the high-risk group had higher M2 macrophage infiltration and that tumor-associated macrophage-related signatures were enriched in this group. Li et al. found that GC-derived mesenchymal stromal cells can induce the polarization of macrophages into the M2 subtype, which promotes the migration and invasion of gastric cancer cells via advancing the process of EMT (64). It has been shown that M2 phenotype polarization of macrophage may contribute to acquired trastuzumab resistance in HER2‐positive GC (65). Thus, we hypothesized that M2 macrophage polarization may contribute to the poor prognosis in high-risk patients.

Drug resistance is a major cause of death in cancer patients (66). We investigated the potential correlation between drug sensitivity and PLT risk scores using CTRP database. The results suggest that patients in the high-risk group tended to be less sensitive to classical antitumor drugs including oxaliplatin, doxorubicin, and mitomycin. The PLT signature may be used as a predictor of tumor response to chemotherapy. On the other hand, we identified three unconventional antitumor compounds including thalidomide, MK-0752, and BRD-K17060750, with potential advantages for patients with high PLT risk scores. Thalidomide combined with capecitabine has been shown in a prior study to be a safe and mildly effective treatment for elderly patients with advanced GC (67). MK-0752 is a potent inhibitor of gamma secretase, an enzyme required for Notch pathway activation. This agent has been investigated in phase 1 clinical trials in solid tumors (68, 69). The efficacy of these drugs for GC patients in the high-risk group is expected for further investigation.

There are some strengths of the present study. Firstly, our signature is based on TCGA data and GEO databases with relatively large sample sizes. Secondly, our risk model is more cost-effective and has good clinical practicability because it’s based on a specific gene set. Thirdly, our risk model and nomogram have great clinical implications for the prognostic evaluation and selection of treatment options for GC patients.

Certain limitations of our study should be addressed. Firstly, more data from prospective clinical GC cohorts need to be collected to externally validate the utility of the model in the next step. Secondly, the public databases provide only a limited amount of information on clinical features and may not include other clinical factors, such as treatment history, and molecular types that can influence prognosis. Lastly, we validated the expression of the risk model genes using qPCR, but further mechanistic studies *in vivo* and vitro need to be conducted to better comprehend the mechanisms by which PLT-related genes affect TME and immunotherapy sensitivity.

In conclusion, our study firstly constructed a reliable PLT-related risk model for predicting survival in GC patients. The independence and predictive performance of this model was further validated using external validation data. This study deepens our understanding of platelet-related genes in GC and provides new potential prognostic and therapeutic biomarkers for individualized treatment.

## Data Availability

The original contributions presented in the study are included in the article/**Supplementary Material**. Further inquiries can be directed to the corresponding author.
