# Supplementary material for: A novel platelets-related gene signature for predicting prognosis, immune features and drug sensitivity in gastric cancer
Source: Front Immunol. 2024 Nov 13;15:1477427. doi: 10.3389/fimmu.2024.1477427 (PMC11599260; doi:10.3389/fimmu.2024.1477427)
Supplement: Supplementary file 2 [file Image1.pdf]

Supplementary Material

1 Supplementary Figures

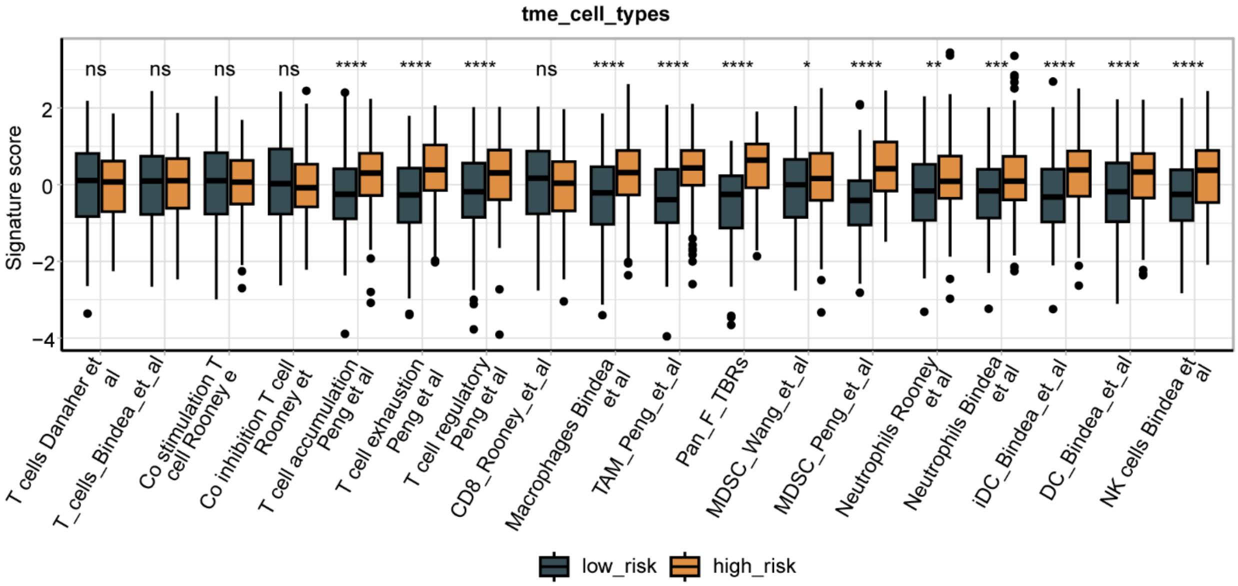

Supplementary Figure S1. Comparison of the enrichment scores of tumor microenvironment cells-related signatures between the high-risk and low-risk groups.

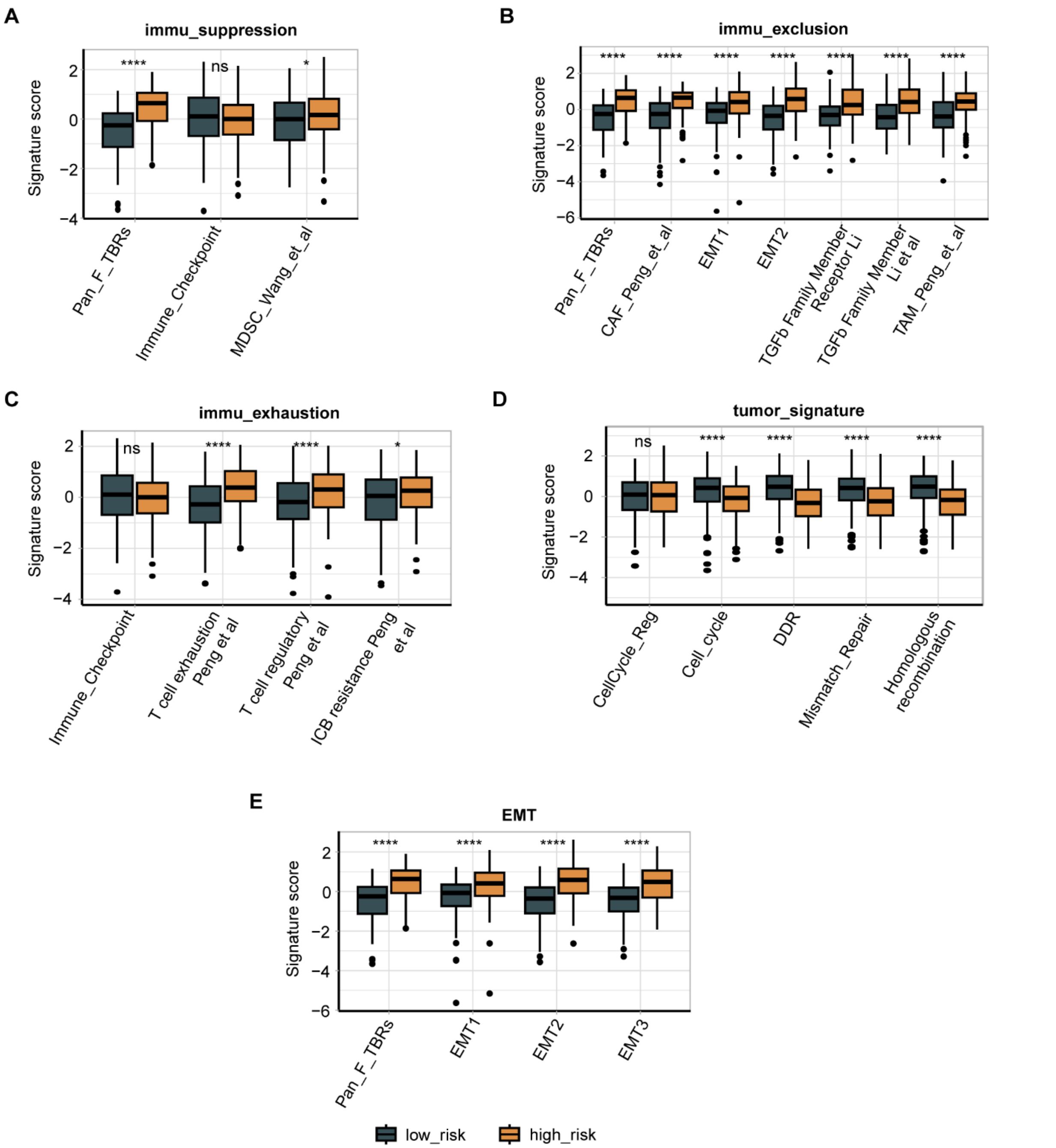

Supplementary Figure S2. Analysis of TME signatures using the IOBR package. **(A-C)** Comparison of immune suppression, exclusion and exhaustion features between two risk groups; **(D)** Comparison of tumor signatures between two groups. **(E)** Comparison of EMT signatures between two groups. *P < 0.05, ****P < 0.0001, ns, not significant.

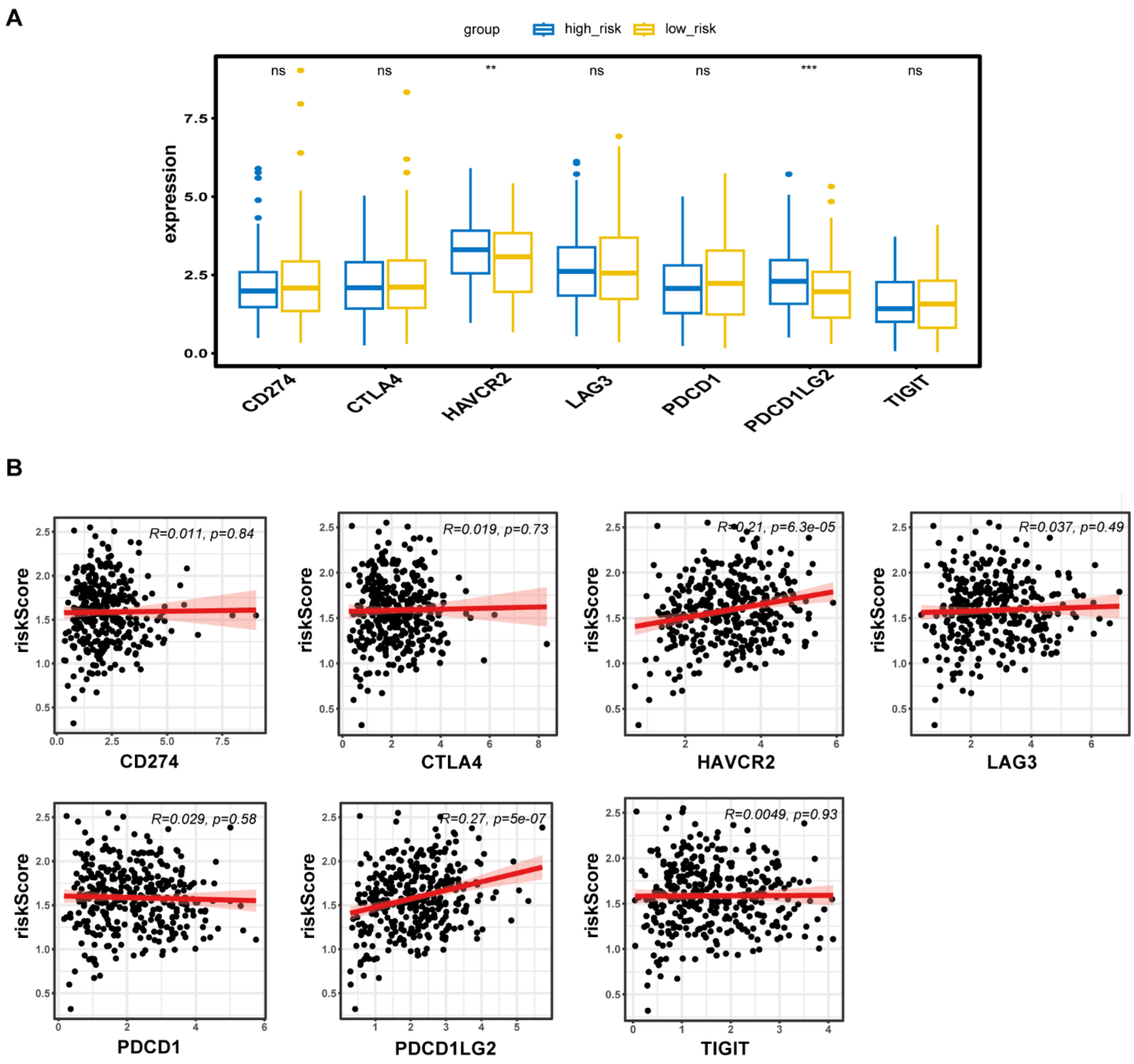

Supplementary Figure S3. Association of risk score and immune checkpoint genes. **(A)** The differential expression of immune checkpoint genes between two risk groups; **(B)** Correlation between immune checkpoint genes and risk score in GC. **P < 0.01, ***P < 0.001, ns, not significant.
